# The impact of dance learning on the social-emotional competence of college students: the mediating role of psychological resilience and the moderating effect of supportive climate

**DOI:** 10.3389/fpsyg.2026.1794754

**Published:** 2026-05-15

**Authors:** Yunyun Xu, Tiefang Liu

**Affiliations:** 1 School of Music and Dance, Hunan First Normal University, Changsha, Hunan, China; 2 School of Educational Science, Hunan Normal University, Changsha, Hunan, China

**Keywords:** dance learning, moderated mediation, psychological resilience, social-emotional competence, supportive climate

## Abstract

**Introduction:**

The development of social-emotional competence has not only become a focus of international attention, but also emerged as a hotspot in current educational psychology research and policy practice worldwide. This study examines the impact of dance learning on social-emotional competence, with a specific focus on the mediating role of psychological resilience and the moderating role of a supportive climate.

**Methods:**

Using an empirical research approach, a cross-sectional survey was conducted among 2,077 university students with 1 year of dance learning experience from 30 higher education institutions across 15 provinces in China. A moderated mediation model was tested using stepwise regression and the PROCESS bootstrap method.

**Results:**

Psychological resilience played a partial mediating role in the relationship between dance learning and social-emotional competence. Supportive climate positively moderated the effects of dance learning on both psychological resilience and social-emotional competence; however, dance learning exerted a significant positive impact on social-emotional competence only in a highly supportive climate. Furthermore, supportive climate positively moderated the indirect effect of dance learning on social-emotional competence through psychological resilience, indicating that the stronger the supportive climate, the more pronounced this indirect effect became.

**Conclusion:**

Establishing a teaching system that cultivates social-emotional competence and achieving its curricular integration has become an urgent need in talent development. This study provides empirical support and practical insights for the assertion that dance teaching is positively associated with the development of college students' social and emotional abilities. These implications not only expand our theoretical understanding of the educational functions of dance art but also offer a scientific basis for educational and instructional reforms at a practical level.

## 1 Introduction

The rapid transformation of the global social environment, escalating academic pressure, and the proliferation of digital socialization pose unprecedented challenges to the mental health and social adaptation of contemporary university students (Zhao and Bai, 2025; Twenge et al., 2018). Extensive research demonstrates a rising prevalence of psychological issues—such as anxiety, depression, and interpersonal difficulties—among the university student population (Auerbach et al., 2016; Sun and Jiang, 2025; World Health Organization, 2022). These issues not only directly impair academic achievement and concentration (Eisenberg et al., 2009) but also exert profound, long-term effects on holistic personal development and career adaptability (Pascoe et al., 2020).

Therefore, exploring positive and effective interventions to promote the holistic development of university students has become an urgent issue for higher education to address. Against this backdrop, social-emotional competencies—defined as a set of core abilities formed through emotional experiences and interactive practices during an individual's socialization process, relating to emotional and social development (Martzog et al., 2016), including the capacity to effectively manage one's own intrapersonal and interpersonal social-emotional experiences (Zimmer-Gembeck and Collie, 2020), have gained increasing prominence. This is because social-emotional competencies have been shown to effectively predict variables such as academic achievement (Durlak et al., 2011), career development, and life satisfaction (Heckman et al., 2006), playing a decisive role in personal success and lifelong wellbeing.

The Organization for Economic Cooperation and Development (OECD) released the *OECD Learning Compass 2030*, identifying social-emotional competencies as one of the essential skills for navigating future changes and positioning them as a key component in the global assessment and monitoring of children and adolescents' capabilities. Numerous international organizations and developed countries have prioritized the enhancement of young people's social-emotional competencies as a critical educational focus. The *Education 2030 Framework for Action* published by UNESCO also explicitly states: “Education must pay greater attention to fostering social-emotional competencies for sustainable development.” It underscores the cultivation of social-emotional competencies as a vital goal and component of school education (Huang, 2022) and integrates it into educational policies (Yao et al., 2023). Consequently, the development of students' social-emotional competencies has not only become a focus of international attention but also a prominent area in contemporary educational psychology research and policy practice worldwide.

As an integral component of art education, dance not only serves the function of aesthetic education but also involves frequent physical activity during the learning process, which helps to release stress and improve mood. The rich interactions involved—such as cooperation, communication, coordination, and empathy—provide an excellent practical setting for individual self-exploration and the development of social skills, offering unique value for cultivating students' psychological resilience and emotional wellbeing (Chen et al., 2025). Existing literature has confirmed the positive psychological impacts of dance learning from various perspectives, demonstrating that it can significantly enhance individuals' social skills, self-confidence, and empathy (Bungay and Vella-Burrows, 2013)—traits that are precisely defined as the core dimensions of social-emotional competence. Concurrently, research in exercise science indicates a close association between regular physical activity and the development of psychological resilience (Liu, 2020). Furthermore, subsequent studies reveal that psychological resilience, acting as a crucial internal psychological resource, exhibits a significant positive predictive effect and a synergistic developmental relationship with the various subsystems of social-emotional competence (McGeown et al., 2017).

Although current studies have formed some independent “isolated islands” of evidence regarding the relationships among these three aspects, few have integrated them within a unified framework to systematically explore their underlying mechanisms of transmission. Specifically, how does dance learning influence social-emotional competencies? What is the internal psychological transformation process? To address this research gap, the core objective of this study is to propose and validate a moderated mediation model aimed at connecting these research “islands,” comprehensively understanding the educational value of dance learning, and providing decision-making references for effective educational practices.

## 2 Literature review and research hypotheses

### 2.1 Dance learning and social-emotional competencies

The Collaborative for Academic, Social, and Emotional Learning (CASEL) delineates social-emotional competencies into five core components: self-awareness, self-management, social awareness, relationship skills, and responsible decision-making. As a multidimensional and embodied learning practice, dance learning exerts positive influences on an individual's physiological, psychological, and cognitive systems, including the development of their social-emotional competencies.

First, dance learning promotes self-awareness and self-management. It requires learners to maintain high attention to their bodily state and movement quality, while also identifying and expressing the various emotions embedded within a dance piece. This process fosters a deeper understanding of their own emotional expression. This sustained awareness of one's internal state (Tang and Huang, 2023) forms the foundation for enhancing self-awareness (Zhang, 2025). Second, dance learning cultivates social awareness and empathy. Dance is essentially a non-verbal dialogue. Learners must learn to “listen” to their peers' body language and respond appropriately. Engaging in processes such as portraying different roles or interpreting dances from diverse cultural backgrounds enables students to transcend an egocentric perspective, better understand others, develop empathy, and embrace diversity (Chakraborty et al., 2024). Consequently, it nurtures the capacity for perspective-taking and understanding others (Liu et al., 2024), thereby substantially exercising an individual's social awareness. Furthermore, dance learning enhances relationship skills. Dance itself is replete with intensive social interactions, including negotiating movement details, resolving conflicts during rehearsals, and offering mutual encouragement and support. These positive verbal and non-verbal communications facilitate the development of social skills in students, such as active listening, negotiation, and collaborative division of labor (Wang and Bu, 2023). Research indicates that students with long-term dance participation demonstrate higher sensitivity in understanding and utilizing non-verbal cues (e.g., facial expressions, body posture), which is a crucial component of communication skills in social interaction (Chakraborty et al., 2024). Finally, dance learning contributes to strengthening responsible decision-making. Within a dance learning team, each member's performance influences the collective outcome. This necessitates that students cultivate a strong sense of responsibility and collective honor. They are required to make responsible decisions, such as arriving punctually for rehearsals, diligently completing their assigned movement parts, and proactively seeking or offering help when encountering difficulties. This commitment to team goals and awareness of the consequences of individual actions are significant manifestations of responsible decision-making ability.

### 2.2 The mediating role of psychological resilience

Psychological Resilience, often understood as an individual's capacity for positive adaptation in the face of adversity, pressure, and challenge (Liu and Feng, 2024), constitutes a comprehensive psychological resource spanning cognitive, emotional, and behavioral dimensions (Hascher et al., 2021). It is not an innate, fixed trait but rather a dynamically evolving capacity influenced by a combination of internal and external factors, which can be enhanced through conscious effort and training. The positive effect of dance learning on the social-emotional competencies of university students is, at least in part, achieved through the enhancement of their psychological resilience.

First, the process of dance learning is filled with challenges, often constituting a “trial-failure-improvement-success” cycle. Within this process, learners must continually confront their own imperfections, analyze the causes of failure, maintain patience and perseverance, adjust their strategies, and try again. This continuous positive feedback loop significantly enhances students‘ self-efficacy and sense of control. Sustained engagement over time helps students cultivate the courage to face difficulties and develop robust stress tolerance (Wang and Bu, 2023), building psychological confidence to meet challenges, which is a key manifestation of high psychological resilience. Furthermore, dance learning provides individuals with a unique, embodied pathway for emotional regulation (Champagne, 2024). When students feel stressed or frustrated, they can release and transform negative emotions through powerful, flowing, or soothing dance movements, rather than merely suppressing or ruminating on them at a cognitive level. This form of emotional expression and management through bodily movement is often more direct and effective than purely verbal or cognitive interventions (Wang and Zhang, 2024). In order to interpret and understand the different emotional states conveyed in dance pieces, students also need to engage positive body postures and emotional memories during the learning process. This practice itself constitutes cognitive reappraisal—re-evaluating and redefining a situation to alter its emotional impact, thereby enhancing university students' psychological flexibility (Dou et al., 2021). This flexibility is a hallmark characteristic of individuals with high psychological resilience. The positive impact of dance on psychological resilience also has a solid psychophysiological foundation. As a form of moderate-to-high-intensity exercise, dance promotes the release of neurotransmitters such as endorphins, dopamine, and serotonin in the brain. These substances effectively improve mood, reduce stress and anxiety, thereby preventing the long-term accumulation of such negative emotions and maintaining a positive emotional state, which serves as an important resource for psychological resilience.

High levels of psychological resilience, cultivated through dance learning and training, have a positive impact on the development of an individual's social-emotional competencies. Firstly, it enables individuals to maintain emotional balance through adaptive emotion regulation strategies, such as cognitive reappraisal (Yu and Liu, 2025). This facilitates superior management of negative emotions, allowing them to maintain a relatively stable emotional state when facing interpersonal conflicts or stressful events. Consequently, emotional dysregulation becomes less frequent, and they are better equipped to express emotions in a mature manner and respond more constructively. Such emotional stability serves as the foundation for establishing and maintaining healthy interpersonal relationships. Furthermore, the enhanced cognitive flexibility fostered by this process helps individuals view problems from multiple perspectives and engage in effective cognitive reappraisal. This ability directly translates into social situations, making them more adept at perspective-taking and understanding others' viewpoints and positions, rather than rigidly adhering to their own. This contributes to demonstrating higher levels of empathy and stronger social awareness (Liu et al., 2024). Concurrently, dance training is demanding and requires long-term persistence. To master a challenging technical movement or complete a full choreographic work, learners must exercise strict self-discipline, manage their frustration, set incremental goals, and exert sustained effort toward them. Each successful attempt significantly bolsters students' self-efficacy and self-esteem. This process itself constitutes an in-depth exercise in developing a positive self-concept and enhancing self-management capabilities.

In summary, when university students participate in dance learning, they are not merely acquiring a set of movements or skills. They are repeatedly practicing coping with setbacks, managing emotions, collaborating with others, and building self-confidence within a safe environment characterized by challenges, demands for persistence, encouragement of cooperation, and allowance for emotional expression. This process subtly yet effectively strengthens their psychological resilience. As psychological resilience is enhanced, students demonstrate a greater capacity for adaptation when confronting pressures and difficulties in their daily academic, personal, and interpersonal lives. They become better at regulating their emotions (self-management), viewing situations more objectively (self-awareness), showing a stronger willingness to understand others (social awareness), and establishing positive relationships with greater confidence (relationship skills). Consequently, the enhancement of psychological resilience acts as a key converter, transforming the external experiential activity of dance learning into stable and efficient internalized social-emotional competencies.

### 2.3 The moderating role of a supportive climate

A supportive climate generally refers to an environment that provides emotional support, encourages positive interactions, and fosters a sense of security for individuals (Jennings and Greenberg, 2009). The development of social-emotional competence relies heavily on substantial social interactions and practical opportunities. Empirical research indicates that when a learning environment exhibits high levels of support, it effectively lowers students' psychological barriers, thereby facilitating the acquisition and internalization of social-emotional skills (Durlak et al., 2011). Because individuals continuously monitor others' feedback during interactions to ascertain the acceptance and validity of their self-presentation (Leary and Kowalski, 1990), a highly supportive climate in dance learning environments makes it easier for students to form positive peer relationships, and such environments effectively facilitate the formation of positive teacher-student interactions (Tsompanaki and Magos, 2022). According to situated learning theory, a dance classroom can be viewed as a “community of practice.” By creating inclusive and encouraging learning contexts, teachers provide a safe space for students to receive feedback from others, ensuring their smooth progression through “legitimate peripheral participation” (Lave and Wenger, 2005). Learning within such a climate provides students with ample opportunities for emotional expression, sharing, seeking understanding, and emotional release. Consequently, they gain more practice and positive feedback in social-emotional skills such as collaboration, communication, and conflict resolution. Empirical studies in performing and arts education have also confirmed that this safe, physically interactive climate can significantly accelerate the development of open-mindedness, emotional regulation, and social interaction skills (Goldstein and Winner, 2012).

Furthermore, a supportive dance learning climate reinforces positive emotional experiences during the learning process. According to the “broaden-and-build” theory, when students perceive support and encouragement from others during dance learning, their positive emotions are amplified. This positive mindset not only counteracts persistent negative emotions but also makes students more willing to attempt novel social behaviors and modes of expression (Fredrickson, 2001). Consequently, they become more engaged in the activity and demonstrate a higher tolerance for mistakes and setbacks (Csikszentmihalyi, 1990), thereby developing their social-emotional competence more effectively. Finally, the impact of dance learning on social-emotional competence requires sustained practice over time; if students withdraw prematurely due to an unsupportive environment, this developmental impact cannot be fully realized. The “need to belong” theory posits that in highly supportive learning environments, students are more likely to develop a robust sense of belonging and self-worth (Baumeister and Leary, 1995). This sense of belonging has been proven to be a powerful protective factor that promotes students' sustained participation in extracurricular activities and ensures the long-term efficacy of educational interventions (Osterman, 2000).

While dance learning itself fosters psychological resilience, a supportive climate can be viewed as a catalyst that accelerates and amplifies this cultivation process. Research indicates that the formation of an individual's psychological resilience is influenced by multiple factors (Daly, 2020), including both intrinsic personal characteristics and external environmental factors (Zheng et al., 2025). Social support constitutes a significant external influence on the development of psychological resilience. In a highly supportive environment, the enhancing effect of dance learning on psychological resilience is likely to be more pronounced. When the environment is filled with support and encouragement, it facilitates the formation of positive teacher-student interactions. These positive interactions can serve as a model for peer interactions (Du and Mao, 2018). Students can promptly acquire coping strategies for stress management, emotion regulation, and problem-solving, as well as emotional solace within these interactions. They are more inclined to mobilize positive psychological resources such as optimism and adopt adaptive behaviors to buffer the impact of stress (Bai and Bai, 2024). All of these factors help students navigate setbacks in dance learning more calmly, adjust their mindset quickly after failure, and try again, thereby reinforcing the development of psychological resilience. Conversely, in a low-support environment, this enhancing effect may be attenuated. When the environment lacks support or even exhibits exclusion and hostility, students are more prone to feelings of loneliness or frustration, leading to a significant reduction in social self-efficacy and proactive social behaviors (Lei et al., 2024). Under such conditions, when stress arises during dance learning, individuals may struggle to effectively alleviate it. Instead, it may trigger avoidance strategies and social withdrawal, thereby weakening relationship-building and conflict management skills. Consequently, the enhancement of psychological resilience is constrained. Therefore, a supportive climate ensures that the challenges inherent in dance learning are transformed into opportunities for building psychological resilience, rather than obstacles that undermine self-confidence.

### 2.4 Research hypotheses

**Figure 1:**
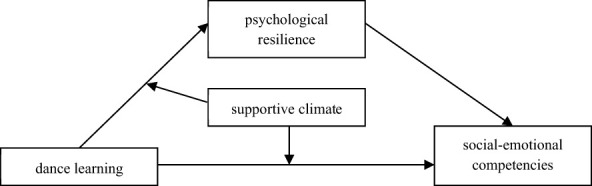
Research model.

Based on previous research and relevant theories, the present study aims to construct a moderated mediation model (see Figure 1) to examine the relationship between dance learning and college students' social-emotional competence, the mediating role of psychological resilience in this relationship, and the moderating role of a supportive climate. Specifically, the following hypotheses are proposed:

H1: Dance learning is positively associated with the development of social-emotional competence among college students;

H2: Psychological resilience mediates the relationship between dance learning and the social-emotional competencies of university students.

H3: A supportive climate positively moderates the impact of dance learning on social-emotional competencies.

H4: A supportive climate positively moderates the impact of dance learning on psychological resilience.

H5: The supportive climate positively moderates the indirect effect of dance learning on social-emotional competencies via psychological resilience. That is, the stronger the supportive climate, the stronger this indirect effect.

## 3 Methods

### 3.1 Participants

The data were collected via an online questionnaire distributed through a third-party survey platform (Wen juan xing). Following the principle of cluster random sampling, university students from 30 higher education institutions across 15 provinces and municipalities, including Beijing, Shandong, Henan, Jiangxi, Fujian, Hunan, Chongqing, Guangdong, and Xinjiang, were recruited for the online survey. The survey was conducted from March to July 2025. A screening question, “Have you participated in dance learning or training in the past year?” was used. Only respondents who answered “yes” proceeded to the main questionnaire; others were excluded. The survey link and QR code generated by the platform were embedded in recruitment materials. The description clearly stated the research purpose, guaranteed anonymity and voluntariness, explained that data would be used solely for academic research, and included an informed consent notice. Clicking “Start Questionnaire” was considered as providing informed consent. Upon completion, invalid questionnaires (e.g., patterned responses, large amounts of missing data, or excessively short completion times) were excluded. Throughout the process, research ethics were strictly adhered to, and no personally identifiable information (e.g., name, ID number) was collected. A total of 2,362 questionnaires were returned. After excluding 285 invalid responses, 2,077 valid questionnaires were retained, yielding a valid response rate of 87.93%. The final sample consisted of 318 males (15.31%) and 1,759 females (84.69%), this proportion reflects the reality that the dance-learning demographic in Chinese universities is predominantly female, rather than being a result of sampling bias. By academic year: 412 freshmen (19.84%), 758 sophomores (36.49%), 713 juniors (34.32%), and 194 seniors (9.35%). Regarding parental education, 649 students (31.24%) had at least one parent with higher education, while 1,428 (68.76%) did not. In terms of major, 1,282 students (61.72%) were from humanities and social sciences, and 795 (38.28%) were from STEM (science, technology, engineering, mathematics) and medical fields.

### 3.2 Research instruments

#### 3.2.1 Dance learning

The measurement of dance learning was adapted from the approach used in the Physical Activity Rating Scale (Liang, 1994). Specifically, the extent of participants' involvement in dance learning and training (including on-campus professional dance courses, extracurricular dance classes, and dance classes at off-campus training institutions) over the past year was assessed across three dimensions: intensity, duration, and frequency. Intensity was measured with the item: “How intense are your dance training and learning sessions? (from light to vigorous).” Duration was measured with the item: “When engaging in dance learning/training at the above intensity, how long does each session typically last? (a. less than 30 min; b. 30 minto 1 h; c. 1 to 1.5 h; d. 1.5 to 2 h; e. more than 2 h).” Frequency was measured with the item: “How often do you engage in such dance learning/training per month? (a. less than once a month; b. 2–3 times a month; c. 1–2 times per week; d. 3–5 times per week; e. almost daily).” All items were scored on a 5-point Likert scale. A composite dance learning score was calculated as: Intensity × Duration × Frequency, reflecting the overall level of dance learning involvement. In this study, the scale demonstrated good internal consistency, with a Cronbach's α coefficient of 0.90.

#### 3.2.2 Social-emotional competencies

Social-emotional competencies were measured using the University Student Social-Emotional Competencies Scale developed by Chen et al. (2023). This scale consists of 26 items across four dimensions. The “Self-Relational Competency” dimension includes 6 items (e.g., “I can overcome difficulties and persist in completing tasks”). The “Other-Relational Competency” dimension includes 5 items (e.g., “I understand the need for perspective-taking and empathize with others' feelings”). The “Collective-Relational Competency” dimension includes 8 items (e.g., “I can uphold collective honor and actively contribute to the group”). The “Responsible Decision-Making Competency” dimension includes 7 items (e.g., “The decisions I make are responsible not only for myself but also for others and the collective”). All items were rated on a 5-point Likert scale, ranging from 1 = “strongly disagree” to 5 = “strongly agree.” In this study, the scale demonstrated high internal consistency, with a Cronbach's α coefficient of 0.93.

#### 3.2.3 Psychological resilience

Psychological resilience was measured using the Chinese version of the Connor-Davidson Resilience Scale (CD-RISC) revised by Yu Xu et al. within the Chinese cultural context (Yu and Zhang, 2007). This scale contains 25 items across three dimensions. The “Tenacity” dimension includes 13 items (e.g., “I do not give up when things seem hopeless”). The “Strength” dimension includes 8 items (e.g., “I try to see the humorous side of things no matter what”). The “Optimism” dimension includes 4 items (e.g., “I try to see the humorous side of things”). All items were rated on a 5-point Likert scale, ranging from 1 = “strongly disagree” to 5 = “strongly agree”. In this study, the scale demonstrated high internal consistency, with a Cronbach's α coefficient of 0.92.

#### 3.2.4 Supportive climate

The supportive climate was measured using an adapted questionnaire. The items were drawn from established Chinese classroom environment assessment tools (Chen and Li, 2009). This scale comprises 20 items across three dimensions. The “Peer Relationship” dimension includes 6 items (e.g., “In dance learning and practice, classmates support and encourage each other”). The “Teacher-Student Relationship” dimension includes 7 items (e.g., “When I encounter difficulties, my teacher helps me”). The “Team Organization” dimension includes 7 items (e.g., “Students actively participate in the affairs and activities of the dance learning team”). All items were rated on a 5-point Likert scale, ranging from 1 = “Always” to 5 = “Never”. Note that for analysis, items were reverse-scored so that higher scores consistently indicated a more supportive climate. In this study, the scale demonstrated high internal consistency, with a Cronbach's α coefficient of 0.91.

#### 3.2.5 Control variables

Variables known from the literature to influence the development of university students' social-emotional competencies, such as family background (Yao and Chen, 2023) and school environmental factors (Loyalka et al., 2021), were included as statistical controls. Specifically, the following demographic variables were measured and controlled for in the analyses: Grade Level, Gender (Coded as 1 for female and 0 for male), Parental Education (Coded as 1 if at least one parent had received higher education and 0 if neither parent had), Academic Discipline (Coded as 1 for students in humanities and social sciences majors and 0 for those in science, technology, engineering, agriculture, or medicine majors).

## 4 Results

### 4.1 Test for common method bias

The measurement variables in this study comprised four constructs, all reported by the same participants, which may lead to concerns regarding common method bias (Podsakoff and Organ, 1986). To assess the impact of such bias, Harman's single-factor test was conducted. The results indicated that, without any rotation and when extracting factors with eigenvalues greater than 1, the first principal component explained only 19.58% of the total variance. This figure did not exceed the commonly used threshold of 40% in academic research (Aulakh and Gencturk, 2000), suggesting that common method bias was not a substantial issue in this study.

### 4.2 Confirmatory factor analysis

To examine the discriminant validity among the variables, confirmatory factor analysis (CFA) was performed using Amos 23.0. The results, presented in Table 1, indicated that the hypothesized four-factor model (dance learning, social-emotional competencies, psychological resilience, and supportive climate) demonstrated a good fit to the data: χ^2^/*df* = 1.863 (<3), RMSEA = 0.065 (<0.08), RMR = 0.031 (<0.05), GFI = 0.913, CFI = 0.907, NFI = 0.938, and TLI = 0.975. Furthermore, the fit indices for this model were significantly better than those for all alternative nested models. These results further suggest that common method bias was not a serious concern in this study and provide evidence for good discriminant validity among the key constructs.

**Table 1:** Results of confirmatory factor analysis for the variables.

Fit index	*χ^2^/df*	GFI	RMSEA	RMR	CFI	TLI	NFI
Four-Factor Model:DS;SC;PR;SE	1.863	0.913	0.065	0.031	0.907	0.975	0.938
Three-Factor Model:DS;SC;PR + SE	2.076	0.872	0.086	0.040	0.756	0.734	0.801
Three-Factor Model:DS + SC;PR;SE	2.549	0.794	0.097	0.045	0.769	0.728	0.796
Two-Factor Model:DS + SC;PR + SE	7.938	0.538	0.158	0.062	0.542	0.674	0.610
Two-Factor Model:DS;SC;PR + SE	8.751	0.517	0.142	0.059	0.536	0.502	0.625
One-Factor Model: DS + SC + PR + SE	15.235	0.579	0.195	0.195	0.560	0.511	0.542

DS, Dance learning; SC, Supportive climate; PR, Psychological Resilience; SE, Social-emotional competence.

### 4.3 Descriptive and correlation analysis

The study employed the Pearson correlation coefficient to examine the relationships between key variables. As shown in Table 2, dance learning was significantly positively correlated with psychological resilience (*r* = 0.582, *p* < 0.001), supportive climate (*r* = 0.215, *p* < 0.05), and social-emotional competence (*r* = 0.667, *p* < 0.001). Psychological resilience was also significantly positively correlated with both supportive climate (*r* = 0.659, *p* < 0.001) and social-emotional competence (*r* = 0.714, *p* < 0.001). Furthermore, social-emotional competence showed a significant positive correlation with supportive climate (*r* = 0.623, *p* < 0.001).

**Table 2:** Correlation analysis of core variables.

Core variables	M	S	1	2	3	4
1. Dance learning	3.177	0.975	1			
2. Psychological resilience	3.425	0.862	0.582^***^	1		
3. Supportive climate	2.719	1.537	0.215^*^	0.659^***^	1	
4. Social-emotional competence	3.683	0.718	0.667^***^	0.714^***^	0.623^***^	1

M, mean; S, standard deviation; *N* = 2077. ^*^ *p* < 0.05, ^**^ *p* < 0.01, ^***^ *p* < 0.001.

### 4.4 Mediation effect analysis

To examine the mediating effect of psychological resilience, this study adopted the procedure proposed by Baron and Kenny (1986). The specific analytical results are presented in Table 3. The results from Model 1 and Model 2 show that dance learning has a significant positive impact on both social-emotional competence (β = 0.356, *p* < 0.001) and psychological resilience (β = 0.533, *p* < 0.001), providing support for Hypothesis H1. In Model 3, after introducing the mediating variable of psychological resilience, psychological resilience shows a significant positive impact on social-emotional competence. Furthermore, the impact of dance learning on social-emotional competence is reduced but remains statistically significant (from 0.356 to 0.155). This indicates that psychological resilience plays a partial mediating role in the relationship between dance learning and social-emotional competence. These findings provide support for Hypothesis H2.

**Table 3:** Results of the analysis on the influence of dance learning and psychological resilience on social-emotional competence.

Variable	Social-emotional competence	Psychological resilience	Social-emotional competence	Social-emotional competence
	Model 1	Model 2	Model 3	Model 4
Constant	5.204^***^	4.276^***^	5.623^***^	6.001^***^
Gender	0.034	−0.005	0.016	0.018
Grade level	0.016	0.103^*^	0.009	0.020
Parental education	0.109^*^	0.057	−0.112	0.087
Academic discipline	0.057	0.026	0.034	0.025
dance learning	0.356^***^	0.533^***^	0.155^*^	0.132^*^
psychological resilience			0.377^***^	
Tenacity				0.562^***^
Strength				0.160^**^
Optimism				0.374^***^
*R^2^*	0.443	0.425	0.525	0.760
*F*	16.729^***^	15.545^***^	19.164^***^	40.321^***^
Δ*R^2^*	0.100	0.225	0.082	0.316

^*^p < 0.05, ^**^p < 0.01, ^***^p < 0.001.

To further examine the mediating role of dance learning on social-emotional competence, this study followed the mediation analysis procedure proposed by Zhao et al. (2010). and conducted a mediation effect test using the Bootstrap method outlined by Preacher and Hayes, 2004 and Hayes (2013). As shown in Table 4, the mediating effect of psychological resilience on the relationship between dance learning and social-emotional competence was significant (LLCI = 0.417, ULCI = 1.413), with a mediate effect size of 0.917. Thus, Hypothesis H2 received further validation.

**Table 4:** Bootstrap analysis of the mediation effect.

Test path	Total effect	Indirect effect	Direct effect	Proportion mediated	95%CI	
					Upper bound	Lower bound
dance learning = > psychological resilience = > social-emotional competence	1.750	0.917	0.8333	52.39%	0.417	1.413

### 4.5 Moderation effect analysis

To test the moderating effect of a supportive climate, this study employed the analytical approach recommended by Ye and Wen (2013). First, the independent and moderating variables were mean-centered. Subsequently, two regression models (Model 2 and Model 4) were established (see Table 5), with psychological resilience and social-emotional competence as the dependent variables, respectively, dance learning as the independent variable, and supportive climate as the moderating variable. The results showed that the regression coefficient for the interaction term between dance learning and supportive climate on psychological resilience was significant (β = 0.322, *p* < 0.001). Similarly, the regression coefficient for the interaction term between dance learning and supportive climate on social-emotional competence was also significant (β = 0.475, *p* < 0.001). This indicates that supportive climate positively moderates the effects of dance learning on both psychological resilience and social-emotional competence. Therefore, Hypotheses H3 and H4 were confirmed.

**Table 5:** Test Results of the moderating effect of supportive climate on the impact of dance learning on psychological resilience and social-emotional competence.

Variable	Model 1	Model 2	Model 3	Model 4
Constant	0.069	0.574^***^	6.001^***^	5.432^***^
Gender	0.040	−0.007	0.017	−0.017
Grade Level	0.001	−0.011	0.023	0.014
Parental Education	−0.033	0.007	−0.050	−0.023
Academic Discipline	0.028	0.015	0.100	0.089
dance learning		0.865^***^		0.655^**^
supportive climate		0.025^***^		0.013
dance learning ^*^ supportive climate		0.322^***^		0.475^***^
*R^2^*	0.200	0.700	0.343	0.533
*F*	6.644^***^	34.277^***^	13.848^***^	16.777^***^
Δ*R*^2^	0.200	0.499	0.343	0.190

^*^p < 0.05, ^**^p < 0.01, ^***^p < 0.001.

**Figure 2:**
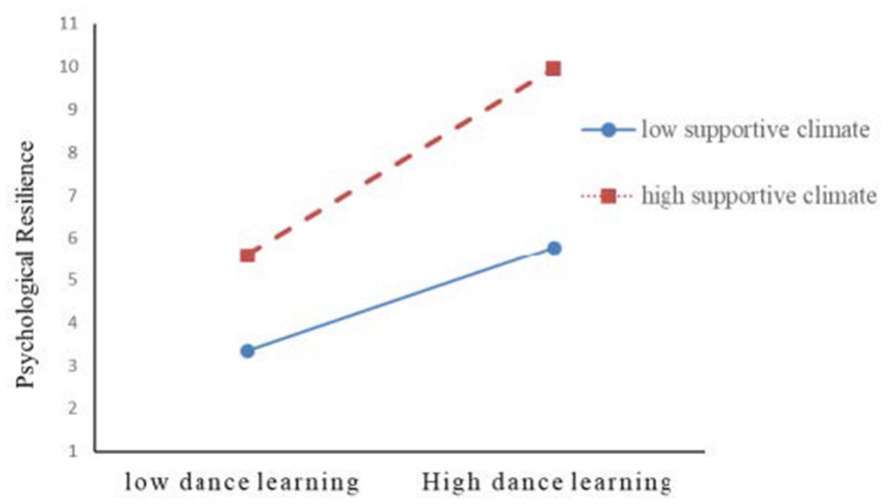
The moderating effect of supportive climate on the relationship between dance learning and psychological resilience.

**Figure 3:**
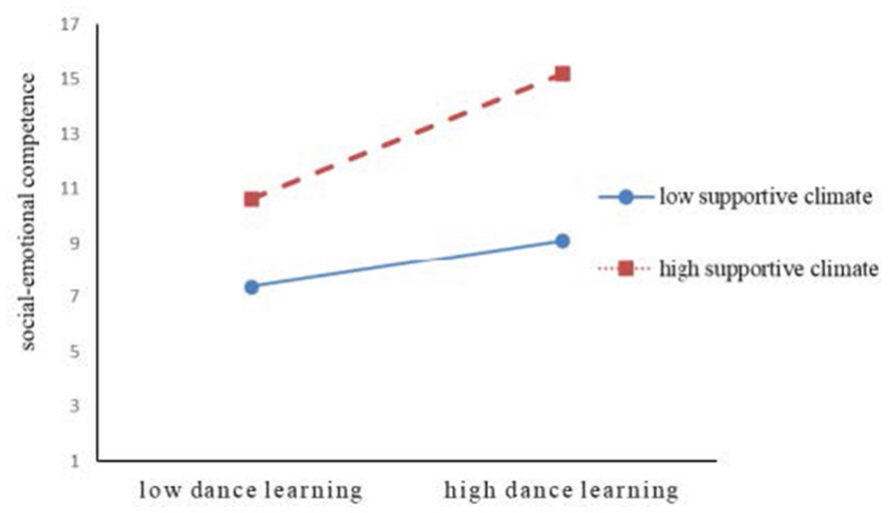
The moderating effect of supportive climate on the relationship between dance learning and social-emotional competencies.

To illustrate the moderation effect patterns, the study plotted the moderation effect graphs following the method recommended by Aiken and West (1991), as shown in Figure 2 and Figure 3. Results of the simple slope analysis (Table 6) revealed that under a low supportive climate, the positive effect of dance learning on psychological resilience was significant [β = 0.807, 95% CI: (0.336, 1.278)], while its positive effect on social-emotional competence was not significant [β = 0.311, 95% CI: (−0.340, 0.961)]. However, under a high supportive climate, the positive effects of dance learning on both psychological resilience [β = 1.340, 95% CI: (0.899, 1.781)] and social-emotional competence [β = 2.259, 95% CI: (1.650, 2.868)] were significant.

**Table 6:** Results of the simple slope analysis.

Dependent variable	Supportive climate	Simple slope	95%CI
Psychological resilience	Low	0.807	[0.336,1.278]
	High	1.340	[0.899,1.781]
Social-emotional competence	Low	0.311	[-0.340,0.961]
	High	2.259	[1.650,2.868]

Having verified the mediating role of psychological resilience and the moderating role of supportive climate in the previous sections, a moderated mediation analysis was conducted to more precisely delineate the relationships among the variables, following the method proposed by Edwards and Lambert (2007). The test results are presented in Table 7.

**Table 7:** Moderated mediation effect analysis.

Mediating variable		Moderating variable		Mediated moderation	
		**Effect**	**(95%CI)**	**Index**	**(95%CI)**
Psychological resilience	Low Supportive Climate	0.140	[−0.137,0.832]	0.035	[0.019,0.134]
	High Supportive Climate	0.597	[0.140,1.168]		
	Difference	0.457	[0.014,0.694]		

The results in Table 7 indicate that in the high-support group, the indirect effect of dance learning on social-emotional competence through psychological resilience is relatively strong [β = 0.597, 95% CI: (0.140, 1.168)]. In contrast, the indirect effect in the low-support group is not significant [β = 0.140, 95% CI: (−0.137, 0.832)]. Moreover, a significant difference exists between the indirect effects at high and low levels of supportive climate, with a difference value of 0.457 [95% CI: (0.014, 0.694)]. The index of moderated mediation was significant (Index = 0.035, 95% CI: [0.019, 0.134]). Specifically, a supportive climate positively moderated the indirect effect of dance learning on social-emotional competence through psychological resilience; that is, the stronger the supportive climate, the stronger this indirect effect. Hypothesis 5 was supported.

## 5 Discussion

This study, employing a moderated mediation model, elucidates the intrinsic mechanism through which dance learning promotes the development of social-emotional competence among university students. It provides a novel theoretical explanation for understanding the complex process of how physical activity is translated into psychological resources and ultimately fosters social adaptation: while dance learning is positively related to the development of university students' social-emotional competence, psychological resilience plays a crucial mediating role, and a supportive climate serves as the boundary condition determining the effectiveness of this positive relationship. This finding not only elevates the understanding of dance's psycho-educational value from descriptive accounts to the level of mechanistic validation but also aligns with contemporary research emphasizing the contextualized and processual nature of competence development. It thereby offers empirical evidence and a pathway reference for higher education institutions to systematically utilize dance art education in fostering students' mental health and personal growth.

### 5.1 The impact of dance learning on university students' social-emotional competence

This study found that college students' participation in dance learning is positively associated with the improvement of their social-emotional competence. This indicates that dance education transcends its traditional “physical education” functions (such as shaping physique and enhancing fitness) and achieves notable “psycho-educational” efficacy. This efficacy is rooted in the micro-social interactive field created by dance learning. Within this field, students continuously accumulate direct social interaction experiences through collaboration, imitation, and emotional resonance with teachers and peers. Simultaneously, dance, as a non-verbal medium for emotional expression, provides students with a unique channel for identifying, regulating, and releasing emotions. These experiences collectively form the practical foundation for the development of an individual's social-emotional competence.

This aligns closely with the “relational perspective” emphasized by Du and Mao (2018), which posits that social-emotional competence is not a static trait but is constructed and developed within specific interpersonal relationships and interactions. The value of this study lies in its specification and empirical validation of dance learning as an effective relational practice for promoting competence development.

### 5.2 The mediating role of psychological resilience

Previous research has largely focused on verifying the direct link between “dance learning” and “positive psychological outcomes.” This study confirms that dance learning not only directly promotes the development of social-emotional competence but also exerts an indirect influence by honing students' psychological resilience, an internal resource (Takahashi and Yamamoto, 2016). The perseverance required to overcome physical limitations during training, the tempering of will through repetitive practice, and the sense of accomplishment from completing a work all provide fertile ground for cultivating psychological resilience. This resilience, fostered through artistic challenge, can be transferred to broader academic and life situations, helping students better manage interpersonal conflicts and cope with academic pressure, thereby exerting a positive impact on the development of their social-emotional competence.

This finding resonates with the “broaden-and-build” theory (Fredrickson, 2001) in positive psychology, which posits that positive dance learning experiences can broaden immediate positive emotions and psychological resources (such as resilience), and these resources can be built into more enduring personal capabilities (such as social-emotional competence) (Lanke and Nath, 2024). Furthermore, this study engages in an interdisciplinary dialogue with conclusions from sports psychology. For instance, Wang and Bu (2023) found that physical exercise influences adolescents' social-emotional competence through the chain mediation of social support and psychological resilience. Our study extends this understanding by independently verifying and emphasizing the centrality of the psychological resilience pathway within the context of dance—an activity that integrates aesthetic and bodily expression—thereby highlighting the unique advantage of dance education in facilitating the transformation of psychological resources.

Importantly, the finding that psychological resilience functioned only as a partial mediator implies the existence of diverse, parallel mechanisms underlying the effect of dance learning on social-emotional competence. Although it represents a central internal transformation pathway, psychological resilience does not preclude the involvement of other latent mediators (e.g., social support, self-efficacy, body image, and self-esteem). Consequently, future research exploring additional parallel or serial mediating pathways is essential to comprehensively uncover the emotional value embedded in dance education.

### 5.3 The moderating role of supportive climate

Previous research has established that the development of social-emotional competence is influenced at multiple levels by the contexts individuals inhabit, the people with whom they interact, and the quality of those relationships (Ju and Guo, 2026). For instance, Yao and Chen (2023) identified school climate as a key factor shaping university students' social-emotional competence.

At a more micro level of individual dance learning, the present study reveals that a supportive climate acts as an “amplifier” in how dance learning affects the development of college students' social-emotional competence. Specifically, while dance learning provides favorable opportunities for social-emotional competence development, translating these opportunities into actual capacity enhancement is not guaranteed. A supportive learning climate emerges as a crucial moderating variable that unlocks the psychological educational function of dance. Only in highly supportive environments does dance learning exert positive effects on both social-emotional competence and psychological resilience.

This is because a climate characterized by teacher encouragement and peer acceptance fosters psychological safety. This safety allows students to transition from a “survival mode”—marked by hiding imperfections, fear of mistakes, avoidance of genuine emotional expression, and a focus on avoiding criticism and failure—to a “learning mode” characterized by courageous exploration and expression. In this mode, students are more willing to help each other, share techniques, provide constructive feedback, and engage in genuine understanding and empathy with their peers, thereby benefiting the development of their social-emotional competence. This echoes the finding of Chen and Li (2009) that students' perceived classroom interpersonal harmony is closely related to their positive social behaviors, which form the foundation for developing robust social-emotional competence through dance.

Furthermore, according to the Transactional Model of Stress and Coping (Lazarus and Folkman, 1984), an individual's appraisal of an event (as a threat or a challenge) determines their subsequent reactions and adaptive outcomes. In dance environments characterized by a lack of support, high competition, and harsh criticism, students are more likely to appraise learning difficulties as threats to their self-worth. When individuals appraise a situation as a “threat” rather than a “challenge,” it triggers negative emotional coping and a lack of psychological safety, subsequently leading to fear and anxiety. To cope with continuous negative evaluations, students are often forced to adopt defensive motivations; their behavioral goals downshift from exploration and expression to merely avoiding mistakes or humiliation. This defensive state is more likely to sever individuals' authentic emotional experiences and expression (Foster, 1997). Students may acquire maladaptive strategies such as emotional suppression, social withdrawal, or self-deprecation, resulting in social isolation and interpersonal problems (Quested and Duda, 2011). In the long term, this can also impair the ability to perceive others' emotions (Gross, 1998), leading to decreased empathy and broken interpersonal trust among peers. This blocks the positive transformation pathway between dance learning and social-emotional experiences, which may explain why the positive effect of dance learning on social-emotional competence tended to be non-significant in low-support environments, as shown in Figure 3 of this study.

Therefore, dance learning itself is not a “safe haven” for socio-emotional development; rather, it exhibits a double-edged sword characteristic that amplifies environmental effects. A lack of support not only negates the educational value of dance but is also likely to turn an activity meant to foster emotional connection into a source of emotional depletion, thereby exerting a substantial negative impact on social-emotional competence. Therefore, the present study provides more granular, process-level evidence supporting the arguments of Ju and Guo (2026), while also highlighting practical directions for maximizing the effectiveness of educational interventions by cultivating a positive classroom climate.

### 5.4 Limitations of the study

This study also has certain limitations. First, regarding data collection. Because this study relies on cross-sectional data for inferential statistics, it is inadequately equipped to establish temporal causal relationships among variables. Furthermore, the exclusive reliance on self-report measures makes it difficult to entirely rule out the influence of common method bias (CMB). Future research could employ longitudinal experimental designs to track the dynamic trajectories of social-emotional competence and psychological resilience in both dance-learning and control groups. By collecting multi-source data, researchers could more robustly establish causal relationships and mitigate the threats of CMB to study validity.

Second, this study did not account for the heterogeneous effects of different dance genres or the influence of long-term dance training backgrounds (e.g., baseline differences between beginners and those with 10+ years of experience), thus lacking an in-depth understanding of how distinct dance styles individually impact cognitive and emotional capabilities. Future studies should explore whether the effects of dance learning on psychological resilience and social-emotional competence vary across different genres, and whether such effects are moderated by long-term dance experience.

Third, the simplified operationalization of “dance learning” constrains the generalizability and interpretation of the current findings. Dance learning is a complex pedagogical and social process (encompassing interaction, expression, and collaboration), and few previous studies have attempted to quantify it. While measuring the extent of dance learning through three dimensions—intensity, duration, and frequency (spanning professional college courses, extracurricular clubs, and off-campus training)—offers strong practical operability, it inevitably oversimplifies the construct's true depth. Future research should refine the operationalization of “dance learning” to ensure that its measurement is grounded in clearer theoretical frameworks.

Collectively, these proposed avenues of research will deepen our understanding of the underlying mechanisms through which dance education fosters college students' social-emotional competence, providing a robust scientific basis for universities to implement more effective dance-based mental health education programs.

## 6 Implications

Currently, establishing a teaching system that cultivates social-emotional competence and achieving its curricular integration has become an urgent need in talent development (Hao et al., 2024). This study provides empirical support and practical insights for promoting the social-emotional competence of university students through dance pedagogy. These implications not only expand our theoretical understanding of the educational functions of dance art but also offer a scientific basis for educational and instructional reforms at a practical level.

### 6.1 Shifting educational philosophy and repositioning the educational value of dance

Previous research has identified the functions of dance learning in “physical education” – shaping the body, improving posture, and forging good physical fitness – and in “aesthetic education” – refining individuals through beauty and enhancing aesthetic appreciation and discernment. This study further demonstrates that dance education is also an indispensable and effective vehicle for “psychological and emotional nurturing.” The findings reveal that dance learning is positively related to the development of college students' social-emotional competence. This challenges the traditional view of dance courses merely as skill training or physical exercise, unveiling their core value in shaping sound character and cultivating psychological literacy. Therefore, it is necessary to re-evaluate and elevate the strategic position of dance arts education in talent cultivation, positioning it as a significant vehicle for promoting students' “whole-person development.”

### 6.2 Shifting teaching philosophy: from “technique-centric” to “student-centered holistic education”

Professional dance courses must not focus solely on the acquisition of specialized skills and the cultivation of aesthetic appreciation. Similarly, public dance courses should not be marginalized as mere “elective hobby classes” or “credits-filling courses.” Their crucial role within the framework of “comprehensive education encompassing moral, intellectual, physical, aesthetic, and labor education” and in fostering students' core competencies must be clearly defined. Dance educators should expand their teaching objectives from the singular goal of “teaching students how to dance” to “facilitating student growth through dance.” They need to recognize their pivotal role in shaping students' psychological resilience and social-emotional competence. For instance, in curriculum design, alongside knowledge and technical objectives, the development of social-emotional competence and the enhancement of psychological resilience should be explicitly included as key pedagogical goals. Teaching segments intentionally designed to strengthen psychological qualities should be incorporated to fully realize the comprehensive educational function of the dance classroom, thereby actively cultivating students' positive psychological attributes. For example, during routine dance feedback, beyond making technical corrections, instructors should intentionally incorporate a “frustration debriefing” segment. This involves encouraging peers to share their psychological shifts when mastering complex and challenging movements, and to offer mutual verbal support. By doing so, physical challenges are transformed into a training ground for resilience, thereby strengthening teacher-student support, fostering self-support, and enhancing psychological resilience to overcome difficulties. Furthermore, in group choreography, educators should shift away from highly controlled “command-style” instruction. Instead, they should design open-ended spatial arrangement tasks, allowing students to cultivate social-emotional competence through the authentic friction of autonomous negotiation and interpersonal conflict resolution.

### 6.3 Fostering a supportive educational environment in dance learning

The study reveals that dance learning can fully realize its function of “psychological and emotional nurturing” only within a supportive climate. Moreover, only in a highly supportive learning climate can the positive impact of dance learning on psychological resilience effectively exert its positive effect on social-emotional competence. This finding underscores that the dance learning environment is not merely a passive backdrop or an inconsequential external condition, but rather a key element influencing the educational efficacy of dance. Therefore, dance instruction should cultivate an open, enjoyable, inclusive, and supportive harmonious climate. The dance classroom should be elevated from a singular venue for skill acquisition to a micro-social field that enriches psychological capital and enhances social- emotional competence. The traditional teaching model focused on movement learning should be developed into an implicit platform for social and emotional learning (SEL), one that boosts students‘ psychological resilience and supports their social development. Admittedly, in low-support instructional environments, students lack the psychological buffer needed to navigate difficulties. Under such circumstances, rather than uncritically advancing technical instruction, teachers must first abandon the authoritarian posture of a “technical referee” and transition into the role of an emotional supporter. By offering genuine acceptance, teachers can establish a foundational sense of psychological safety for students. Furthermore, through the innovation of micro-interaction strategies, they can ultimately evolve into “icebreakers” that catalyze the growth of students' psychological resilience, and “weavers” of robust social support networks.

### 6.4 Enriching the educator's role and expanding the core competency requirements for dance teachers

The shift in dance education philosophy and teaching models necessitates that dance teachers re-examine their roles. In the dance classroom, a teacher's primary task may no longer be merely transmitting knowledge or skills, but rather becoming a creator and sustainer of a positive, supportive learning environment. Each word of encouragement, every instance of patient guidance, and every act of empathizing with and accepting students' emotions serve as a “catalyst” for the transformation of student capabilities. This requires that teacher training and evaluation systems place high importance on the ability to foster a supportive classroom climate, recognizing it as one of the core competencies for dance teachers.

## 7 Conclusions

The study yielded several key findings. First, dance learning is significantly and positively associated with both social-emotional competence and psychological resilience. Second, psychological resilience partially mediates the relationship between dance learning and social-emotional competence. Third, a supportive climate positively moderates these associations. Specifically, the positive association between dance learning and psychological resilience remains significant regardless of the climate level; however, the positive association between dance learning and social-emotional competence is only significant under a high supportive climate. Finally, a supportive climate positively moderates the indirect association of dance learning with social-emotional competence via psychological resilience, with this indirect relationship strengthening as the supportive climate increases.

## Data Availability

The raw data supporting the conclusions of this article will be made available by the authors, without undue reservation.
